# IL-10-Producing B Cells Are Induced Early in HIV-1 Infection and Suppress HIV-1-Specific T Cell Responses

**DOI:** 10.1371/journal.pone.0089236

**Published:** 2014-02-21

**Authors:** Jun Liu, Wei Zhan, Connie J. Kim, Kiera Clayton, Hanqi Zhao, Erika Lee, Jin Chao Cao, Blake Ziegler, Alexander Gregor, Feng Yun Yue, Sanja Huibner, Sonya MacParland, Jordan Schwartz, Hai Han Song, Erika Benko, Gabor Gyenes, Colin Kovacs, Rupert Kaul, Mario Ostrowski

**Affiliations:** 1 Clinical Sciences Division, University of Toronto, Toronto, Ontario, Canada; 2 Department of Immunology, University of Toronto, Toronto, Ontario, Canada; 3 Maple Leaf Clinic, Toronto, Ontario, Canada; 4 Li Ka Shing Knowledge Institute, St. Michael’s Hospital, Toronto, Ontario, Canada; New York University, United States of America

## Abstract

A rare subset of IL-10-producing B cells, named regulatory B cells (Bregs), suppresses adaptive immune responses and inflammation in mice. In this study, we examined the role of IL-10-producing B cells in HIV-1 infection. Compared to uninfected controls, IL-10-producing B cell frequencies were elevated in both blood and sigmoid colon during the early and chronic phase of untreated HIV-1 infection. *Ex vivo* IL-10-producing B cell frequency in early HIV-1 infection directly correlated with viral load. IL-10-producing B cells from HIV-1 infected individuals were enriched in CD19^+^TIM-1^+^ B cells and were enriched for specificity to trimeric HIV-1 envelope protein. Anti-retroviral therapy was associated with reduced IL-10-producing B cell frequencies. Treatment of B cells from healthy donors with microbial metabolites and Toll-like receptor (TLR) agonists could induce an IL-10 producing phenotype, suggesting that the elevated bacterial translocation characteristic of HIV-1 infection may promote IL-10-producing B cell development. Similar to regulatory B cells found in mice, IL-10-producing B cells from HIV-1-infected individuals suppressed HIV-1-specific T cell responses *in vitro*, and this suppression is IL-10-dependent. Also, *ex vivo* IL-10-producing B cell frequency inversely correlated with contemporaneous *ex vivo* HIV-1-specific T cell responses. Our findings show that IL-10-producing B cells are induced early in HIV-1 infection, can be HIV-1 specific, and are able to inhibit effective anti-HIV-1 T cell responses. HIV-1 may dysregulate B cells toward Bregs as an immune evasion strategy.

## Introduction

Regulatory B cells (Bregs, also called B10s) are a rare subset of B cells producing IL-10 that was recently identified in mice and humans [1]–[5]. Bregs suppress autoimmune diseases through inhibiting self-reactive CD4^+^ T cells [1], [2], [4]–[8]. Bregs have been shown to suppress immune responses against pathogens and tumors in mice [9]–[13]. Notably, hepatitis B virus (HBV)-specific CD8^+^ T cell responses in chronic HBV infected individuals were suppressed by Bregs [14]. Suppression is predominantly IL-10 mediated [1], [2], [4], [5], [10]–[12], [14]. The mechanisms that regulate Breg genesis and function are not clear yet, but various molecules, including TLR ligands, CD154 (CD40L), foreign antigens, and IL-21, were shown to promote differentiation of B cells to Bregs by signaling through cognate receptors on B cells [2], [8], [15].

Human Immunodeficiency Virus Type 1 (HIV-1) infection is a chronic persistent infection for all individuals infected, despite the detection of strong T cell responses early in infection, which can partially control virus replication [16]–[19]. Virus persistence is associated with dysfunctional T cell responses [20]–[22]. HIV-1-specific CD4^+^ T cell responses are rapidly eliminated or dysfunctional early in infection in the majority of individuals [19], [23] and the HIV-1-specific CD8^+^ cytotoxic T cell (CTL) response develops functional abnormalities typical of T cell exhaustion during persistent viremia [24]–[26]. HIV-1 infection is also associated with various anomalies in B cells [27], including aberrant polyclonal B cell activation resulting in increased levels of polyclonal immunoglobulins and auto-antibodies, and impairment in neoantigen and recall antigen B cell responsiveness [28]–[31]. This is associated with a contraction in naïve and memory B cell populations and an expansion of apoptosis-prone immature transitional CD10^+^CD27^−^ B cells and mature activated CD21^lo^CD10^−^ B cells [32]–[35]. This milieu may prevent the rapid development of an effective neutralizing antibody response to HIV-1. Given the role of IL-10-producing Bregs in microbial persistence [10]–[14] and a previous report that IL-10 mRNA transcript was upregulated in peripheral blood B cells in HIV-1 infected individuals [36], we investigated the role of IL-10-producing B cells in HIV-1 infection as a potential immune evasion strategy. Since the term Bregs is used to denote IL-10-producing B cells with suppressive function [37], and B10 is used for Bregs producing IL-10 after *ex vivo* phorbol-12-myristate-13-acetate (PMA) plus ionomycin stimulation [3], [7], [8], for clarity and consistency we use the term IL-10-producing B cells in this manuscript to denote B cells producing IL-10 constitutively or after *ex vivo* PMA/ionomycin stimulation.

## Materials and Methods

### Subjects

All subjects were recruited under a protocol approved by the ethics committee at St. Michael’s hospital, Toronto, an affiliate of the University of Toronto. Written consent was obtained from all participants. HIV-1 infected individuals were grouped as follows: a) untreated early infection (EI) (n = 25, not all samples were used in each experiment): positive HIV-1 EIA and HIV-1 western blot with negative HIV-1 EIA within the previous 6 months without anti-retroviral treatment (ART) (mean CD4^+^ T cell count = 561/mm^3^ (range 290–870) and mean viral load = 32,535 RNA copies/mL (range 375–225,590)); b) untreated chronic infection (CI) (n = 15, not all samples were used in each experiment): infected for more than 1 year without prior ART (mean CD4^+^ T cell count = 360/mm^3^ (range 210–960) and mean viral load = 97,290 RNA copies/mL (range 2714–500,001)); c) treated chronic infection (TCI) (n = 10): infected for more than 1 year before going on more than 1 year of continuous ART before sampling (mean CD4^+^ T cell count = 661/mm^3^ (range 340–1820) and mean viral load = 326 RNA copies/mL (range 49–2420)); d) long-term nonprogressors (LTNP) (n = 6): asymptomatic, untreated HIV-1 infection for more than 10 years with CD4^+^ T cell count above 500/mm^3^ and viral load below 10,000 RNA copies/mL (mean CD4^+^ T cell count = 779/mm^3^ (range 530–1024) and mean viral load = 337 RNA copies/mL (range 49–1577)).

### Sample Preparation

Peripheral blood mononuclear cells (PBMCs) were isolated from leukopheresis samples with standard Ficoll-Hypaque procedure and frozen immediately at −150°C until use. Fresh PBMC was used for some of the experiments. Single cells were prepared from sigmoid colon biopsy samples as reported previously [38]. Briefly, colonic biopsies were digested for two rounds in Collagenase type II (Sigma, St. Louis, MO) at 0.5 µg/ml and then 1.0 µg/ml on a shaking heating block at 37°C for 30 min each. The cell suspension was then filtered through a 100-µm filter and enumerated.

### Cell Isolation

T and B cells were isolated using Human T Cell or B cell Negative Selection Kit (Stemcell, Vancouver, Canada). The purity of T and B cells was >96%. For isolation of TIM-1^+^ B and TIM-1^−^ B cells, purified B cells were stained with phycoerythrin (PE) mouse anti-human TIM-1 mAb (Biolegend, San Diego, CA) for 30 min. Then TIM-1^+^ B cells were isolated using PE Selection Kit (Stemcell) and the remaining B cells were TIM-1^−^ (purity>95%).

### Flow Cytometry

The following anti-human monoclonal antibodies were used: IL-10, CD1d, CD3, CD4, CD5, CD19, CD20, CD24, CD27, CD38, TNF-α (eBioscience, San Diego, CA); CD10, CD21, CD107a (Biolegend); TIM-1 (Abcam, Cambridge, MA); IFN-γ (BD Biosciences, San Jose, CA); CD8 (Invitrogen). Purified human IgG (Biolegend) was used to block Fc receptors. For IL-10-producing B cell identification, unstimulated PBMCs or PBMCs stimulated with phorbol-12-myristate-13-acetate (PMA, Sigma) plus ionomycin (Sigma) were cultured in medium in the presence of GolgiStop and GolgiPlug (BD Bioscience) for 5 h and then stained with IL-10, CD3, and CD19 antibodies. For gp140-staining of B cells, PBMCs were first stained with 20 µg/mL of trimeric HIV-1 YU2 gp140 with FLAG-tag at N-terminus, and then stained with FITC-anti-FLAG mAb (Sigma) and CD3, CD19, and TIM-1 antibodies. The trimeric HIV-1 YU2 gp140 is a fusion construct of an N-terminal FLAG tag, a codon-optimized and uncleaved HIV-1 YU2 gp140, and a C-terminal trimerization domain foldon from T4 bacteriophage fibritin [39], which was expressed in HEK-293T cells and purified with FLAG-tag affinity purification [40]. The purified trimeric HIV-1 YU2 gp140 was predominantly trimer and not monomer or dimer as verified by Native-PAGE and could be immunoprecipitated by a broadly neutralizing mAb b12 that can recognize HIV-1 Env native spike [40]. For cytokine production and degranulation of T cells, we used Blair et al.’s protocol [1] with modification: purified T cells were co-cultured with purified TIM-1^+^ B cells or TIM-1^−^ B cells at 1∶1 ratio in a 96-well plate pre-coated with anti-CD3 (Biolegend) for 3 d and then stimulated with HIV-1 p55 (Austral Biologicals, San Ramon, CA) and 15-mer Gag pool (NIH AIDS Reagent Program, Bethesda, MD) or cultured in medium in the presence of GolgiStop and GolgiPlug for 6 h and then stained with various antibodies. In some experiments, recombinant human IL-10 receptor alpha subunit (R&D, Minneapolis, MN) was used for depleting IL-10 in the T+TIM-1^+^ B cell co-culture. For T cell proliferation, CFSE-labeled PBMCs were stimulated with HIV-1 Gag peptide pool or cultured in medium for 6 d and then stained with CD3^+^, CD4^+^, and CD8^+^ mAbs. Proliferation of T cells was calculated as the percentage of CFSE_lo_ T cells in T cells stimulated with HIV-1 Gag peptide pool subtracting the background in T cells cultured in medium. Isotype or FMO (Florescence minus one) control was used as gating control in flow cytometry analysis.

### Luminex Assay

Secreted IL-10 from purified B cells was quantitatively measured by IL-10 Luminex (EMD Millipore, Etobicoke, Canada). 10^5^ purified B cells were plated into the lower chamber of a 96-well transwell plate (Corning, Tewksbury, MA). For stimulation, TLR agonists were added to the B cells. Human IL-10 capture beads were added to the upper chamber of the 96-well transwell plate. 12 h later, the beads were harvested, washed and read according to manufacturer’s protocol.

### Statistical Analysis

Kruskal-Wallis one-way analysis of variance (ANOVA) was used for comparisons between multiple groups and then Dunn’s test was used for pair-wise comparisons. Wilcoxon rank-sum test was used for comparison between two groups. Wilcoxon matched pairs test was used for comparison of two groups’ data from paired individuals. Pearson’s correlation was used for correlation analysis. All statistical analyses were done using Prism (GraphPad Software). *P*<0.05 was considered significant.

## Results

### IL-10-producing B Cells are Elevated in Peripheral Blood of Untreated HIV-1 Infected Individuals

**Figure 1:**
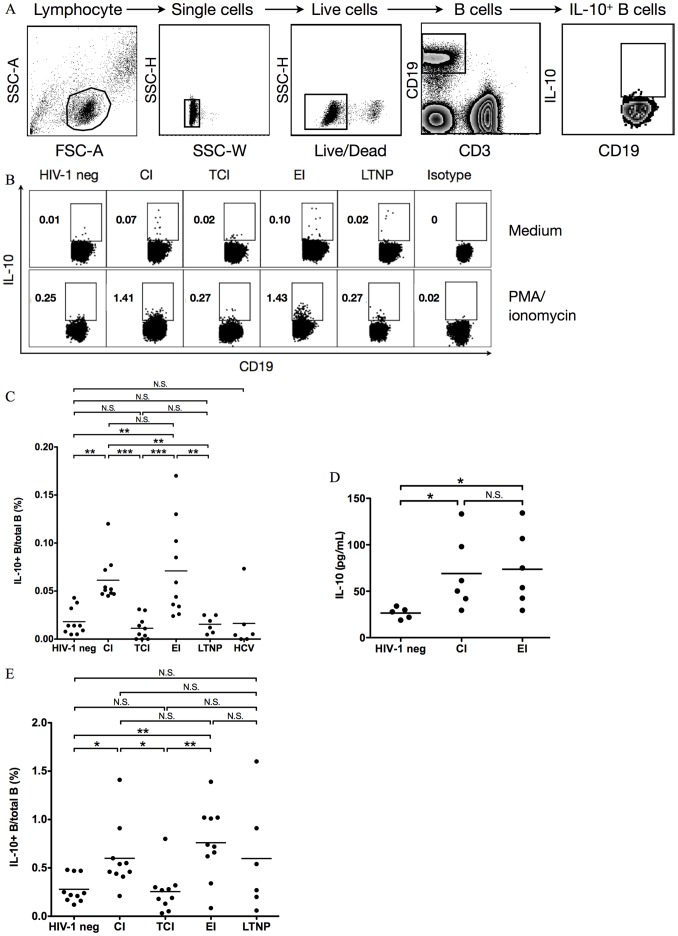
IL-10-producing B cell frequency is elevated in PBMC of untreated HIV-1 infection. (A) Gating strategy for identification of *ex vivo* IL-10-producing B cells in PBMC. (B) Flow cytometry dot plots of IL-10-producing B cells in unstimulated PBMC and PMA/ionomycin-stimulated PBMC from representative HIV-1 infected individuals and a healthy donor. (C) Summary data of IL-10-producing B cell frequencies in unstimulated PBMC from a cohort of HIV-1 infected individuals at different stages, healthy donors, and HCV mono-infected individuals. Each circle represents one individual. (D) IL-10 production from purified B cells of healthy donors and untreated HIV-1 infected individuals. B cells were purified from PBMC and cultured in unstimulated medium for 12 h and then IL-10 in the supernatant was measured by a Luminex assay. (E) Summary data showing IL-10-producing B cell frequency in PBMC stimulated 5 h with PMA/ionomycin from healthy donors and HIV-1 infected individuals. HIV-1 neg: healthy donors. CI: untreated chronic HIV-1 infection. TCI: treated chronic HIV-1 infection. EI: untreated early HIV-1 infection. LTNP: long-term non-progressors. HCV: HCV mono-infection. Isotype: representative IL-10 antibody isotype control from the same EI patient shown in Figure 1B. *: *P*<0.05. **: *P*<0.01. ***: *P*<0.001. N.S.: non-significant (Kruskal-Wallis one-way ANOVA and Dunn’s test). For flow cytometry analysis, >1.5 million events in the lymphocyte gate were collected.

*Ex Vivo* IL-10-producing B cells are detected at low frequencies in PBMC from healthy subjects [3], [14], however, they are elevated in chronic HBV [14]. We first examined the frequency of IL-10-producing B cells directly *ex vivo* in unstimulated PBMC from HIV-1 infected individuals and healthy blood donors. The frequency of IL-10-producing B cells in unstimulated PBMC was very low (0.019%±0.004% in B cells, mean±s.e.m., n = 10) in healthy donors, consistent with a previous report [14] (Figure 1). IL-10-producing B cell frequencies were elevated in untreated early infection (EI) (0.072%±0.016%, n = 10) and untreated chronic infection (CI) (0.061%±0.007%, n = 10) but not in chronically infected individuals on treatment for >1 year (TCI) (0.011%±0.004%, n = 10) or long-term nonprogressors (LTNP) (0.015%±0.004%, n = 6) (Figure 1B and 1C). We did not detect significantly elevated IL-10-producing B cell frequencies in unstimulated PBMC from individuals mono-infected by hepatitis C virus (HCV) with detectable viremia (mean viral load = 3,436,000 IU/mL, range 27000–8,060,000 IU/mL, n = 6) (Fig. 1C). These observations were confirmed by utilizing a different assay, in which *ex vivo* purified B cells were cultured in medium for 12 h and examined for IL-10 secretion by luminex assay (Figure 1D).

Previous work has shown that a short pre-stimulation of PBMC with PMA and ionomycin can enhance the detection of IL-10-producing B cells, which were called ‘B10 cells’ that potently suppressed T cell responses [3], [7], [8]. We similarly determined the frequency of IL-10-producing B cells after PMA/ionomycin pre-stimulation in our cohort. As shown in Figure 1E, we could enhance the detection of IL-10-producing B cells after pre-stimulation in peripheral blood from healthy donors (0.28%±0.044% in B cells, n = 10) by about 15-fold compared to unstimulated PBMC (Figure 1B, 1C and 1E). Moreover, pre-stimulation enhanced the detection of IL-10-producing B cells in EI and CI individuals that were significantly elevated compared to TCI and healthy donors (Figure 1E). IL-10-producing B cells after pre-stimulation were not significantly elevated in LTNP compared to healthy donors, although some LTNP did have elevated IL-10-producing B cells frequencies by this assay. Under both directly *ex vivo* and PMA/ionomycin stimulation conditions, we did not find significantly different IL-10-producing B cell frequencies between EI and CI individuals. Taken together, these results indicate that IL-10-producing B cell frequencies are elevated in untreated viremic HIV-1 infection.

### The Frequency of IL-10-producing B Cells Correlates with Viral Load in Early HIV-1 Infection

**Figure 2:**
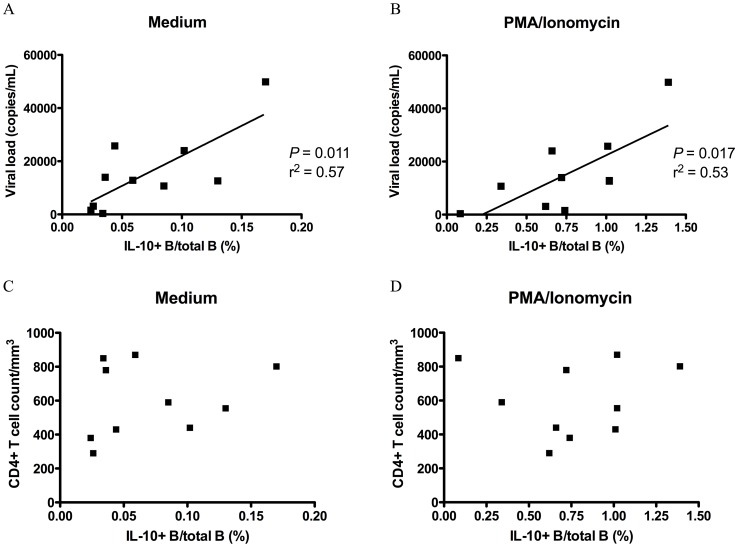
Correlation of IL-10-producing B cell frequency with HIV-1 plasma viral load and CD4^+^ T cell count in untreated early HIV-1 infection. Correlation of IL-10-producing B cell frequency in (A) *ex vivo* unstimulated PBMC and (B) PBMC stimulated with PMA/ionomycin for 5 h with viral load. Correlation of IL-10-producing B cell frequency in (C) unstimulated PBMC and (D) PBMC stimulated with PMA/ionomycin for 5 h with CD4^+^ T cell count. Ten HIV-1 EI subjects were tested. Pearson’s correlation was used for correlation analysis.

To evaluate how IL-10-producing B cell frequency was associated with HIV-1 disease progression, we performed a correlation analysis of IL-10-producing B cell frequency with HIV-1 viral load and CD4^+^ T cell count. IL-10-producing B cell frequency measured either directly *ex vivo* or after PMA/ionomycin stimulation directly correlated with viral load but not with CD4^+^ T cell count in EI (Figure 2). In CI, no such correlation was found (Figure S1) (see Discussion).

### IL-10-producing B Cells in HIV-1 Infection are Enriched in TIM-1^+^ B Cells

**Figure 3:**
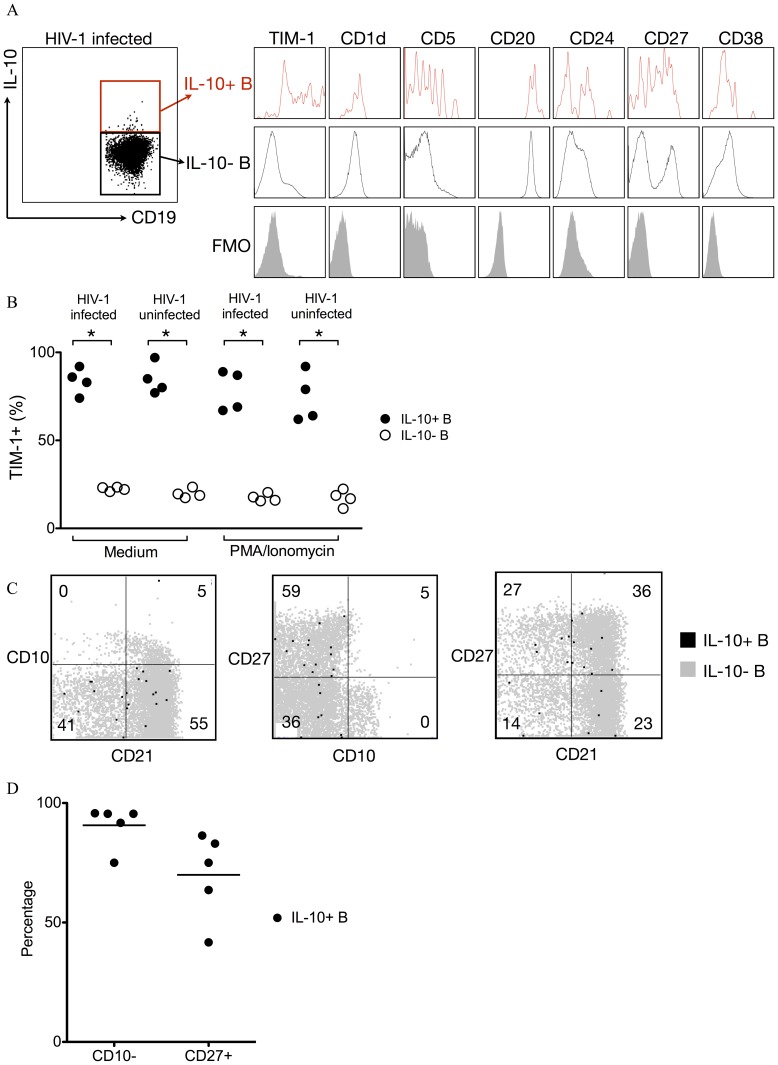
Phenotype of IL-10-producing B cells in HIV-1 infection. (A) Representative phenotype of IL-10-producing B cells in the unstimulated PBMC of HIV-1 viremic individuals. (B) TIM-1 expression on IL-10-producing B cells and non-IL-10-producing B cells in unstimulated PBMC (Medium) or PBMC stimulated 5 h with PMA/ionomycin (PMA/Ionomycin) of eight individuals (4 healthy donors, 2 HIV-1 infected EI subjects, and 2 HIV-1 infected CI subjects). Filled shapes represent IL-10-producing B cells and open shapes represent non-IL-10-producing B cells. *: *P*<0.05 (Wilcoxon rank-sum test). (C) Representative activation/maturation marker expression of IL-10-producing B cells (black dots) in the unstimulated PBMC of HIV-1 viremic individuals overlaying on total B cells (grey dots). Numbers in each quadrant indicate the percentage of IL-10-producing B cells bearing the quadrant’s phenotype. (D) Percentage of IL-10-producing B cells exhibiting the CD10^−^ phenotype or the CD27^+^ phenotype. Shown is the summary from 5 HIV-1 viremic individuals.

Various surface markers have been used to identify or isolate Bregs, though none of them are exclusively IL-10-producing B cell specific [37]. Previous reports showed that human IL-10-producing B cells are enriched in CD24^hi^CD38^hi^ immature-like [1] or CD24^hi^CD27^+^ memory-like B cells [3]. We analyzed surface molecules expressed on IL-10-producing B cells in unstimulated PBMC of HIV-1 infected individuals and found they were CD19^+^CD20^+^TIM-1^+^CD1d^hi/lo^CD5^+/−^CD24^hi/lo^CD27^+/−^CD38^hi/lo^ (Figure 3A). Recently, mouse Bregs were found to be preferentially enriched with TIM-1^+^ B cells than with another proposed phenotype of CD1d^hi^CD5^+^ B cells [41]. Furthermore, engagement of TIM-1 on mouse B cells was shown to promote B cells to differentiate into Bregs [41], [42]. We found that compared with B cells not producing IL-10, IL-10-producing B cells were enriched in TIM-1^+^ B cells (Figure 3A and 3B). TIM-1 was expressed on most IL-10-producing B cells both directly *ex vivo* (84.3%±2.7%) or after PMA/ionomycin stimulation (76.1%±4.3%), while most B cells not producing IL-10 were TIM-1^−^ without or with PMA/ionomycin stimulation (78.9%±0.8% and 82.6%±1.2%, respectively) (Figure 3B). For all samples studied, IL-10-producing B cells were significantly enriched in TIM-1^+^ B cells vs. TIM-1^−^ B cells (*P*<0.05, Figure 3B). Thus, TIM-1 is a preferentially expressed surface marker for IL-10-producing B cells.

Using CD10/CD21/CD27, Moir et al. categorized peripheral blood B cells into CD10^+^CD27^−^ immature/transitional, CD21^lo^CD10^−^ activated/mature, CD21^lo^CD27^−^ exhausted tissue-like memory and CD21^hi^CD27^+^ resting/memory subsets and found an expansion of the former three subsets and a contraction of the latter in HIV-1 viremic individuals [43]–[45]. As shown in Figure 3C and 3D, we found that most IL-10-producing B cells detected directly *ex vivo* in unstimulated PBMC from HIV-1 viremic individuals were CD10^−^ (90.68%±3.991%) and CD27^+^ (69.94%±8.077%), indicating IL-10-producing B cells in HIV-1 viremic individuals tend to be of memory but not of transitional nor exhausted phenotype.

### IL-10-producing B Cells in HIV-1 Infection can be HIV-1 Specific

**Figure 4:**
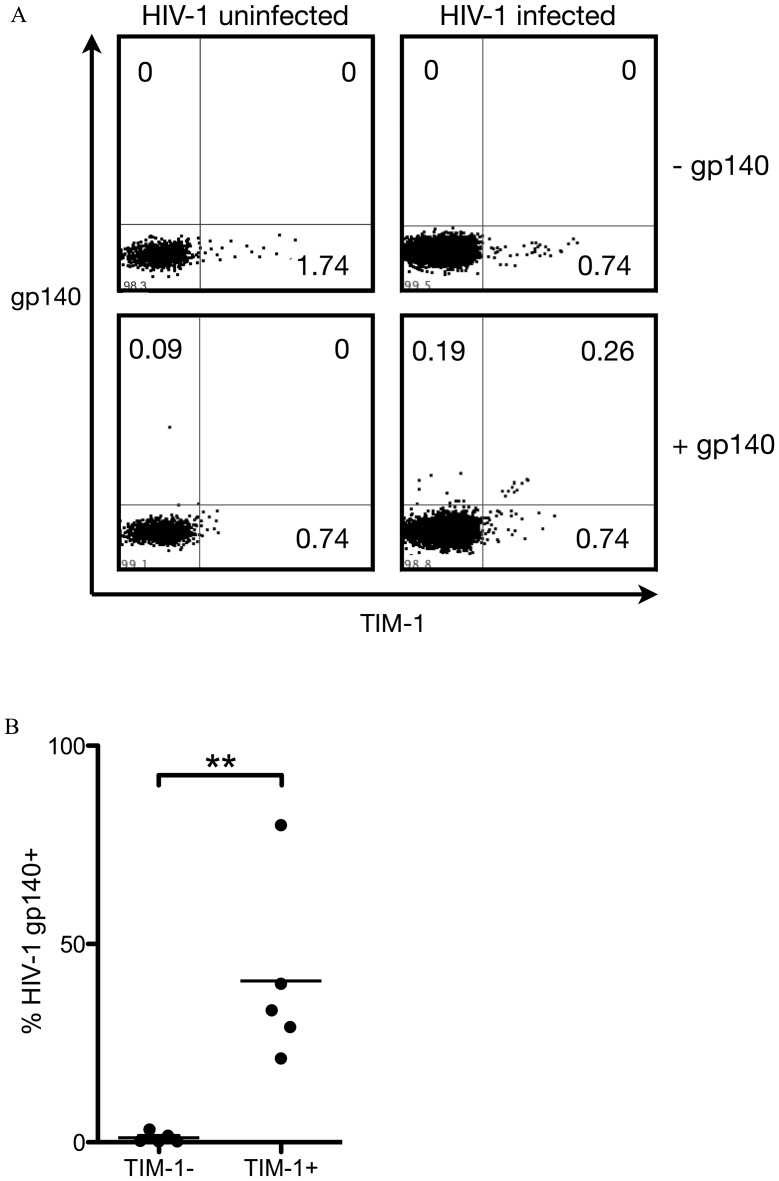
TIM-1^+^ B cells were enriched with HIV-1 trimeric Env-specific B cells. PBMCs were incubated with a FLAG-tagged HIV-1 YU2 gp140 trimer (see Materials and Methods) and then stained with anti-FLAG mAb, CD3, CD19, IgD, and TIM-1 mAbs. (A) Representative flow cytometry dot plot showing gp140 and TIM-1 co-staining of IgD^−^ B cells from an HIV-1 viremic individual. Staining of the same HIV-1 viremic individual without gp140 trimer (No gp140) and staining of an HIV-1 uninfected subject (HIV-1 uninfected) were used as gating controls for gp140 gating. (B) Summary of gp140 staining on TIM-1^+^ IgD^−^ and TIM-1^−^ IgD^−^ B cells from five HIV-1 viremic individuals. **: *P*<0.01. (Wilcoxon rank-sum test).

Neutralization activity of HIV-1 neutralizing antibodies is associated with their binding to soluble trimeric HIV-1 envelope protein (Env), which was assumed to mimic the native conformation of Env on HIV-1 virions [46]. To delineate if IL-10-producing B cells in HIV-1 infected individuals were specific for trimeric Env, we stained B cells with a recombinant trimeric HIV-1 gp140 and used TIM-1 as a marker for IL-10-producing B cells. Representative data from one HIV-1 infected individual and one uninfected individual are shown in Figure 4A and summary data from infected individuals are shown in Figure 4B. TIM-1^+^ B cells were enriched for HIV-1 trimeric Env specificity compared with TIM-1^−^ B cells, though the enrichment was variable between different HIV-1 infected individuals (21%–80% of TIM-1^+^ B cells were positively stained by trimeric HIV-1 gp140). Thus, IL-10-producing B cells in HIV-1 infection are enriched for HIV-1 trimeric Env specificity.

### IL-10-producing B Cells Exhibit Regulatory Activity by Suppressing HIV-1-specific Cytokine Production/Degranulation of T Cells in vitro

**Figure 5:**
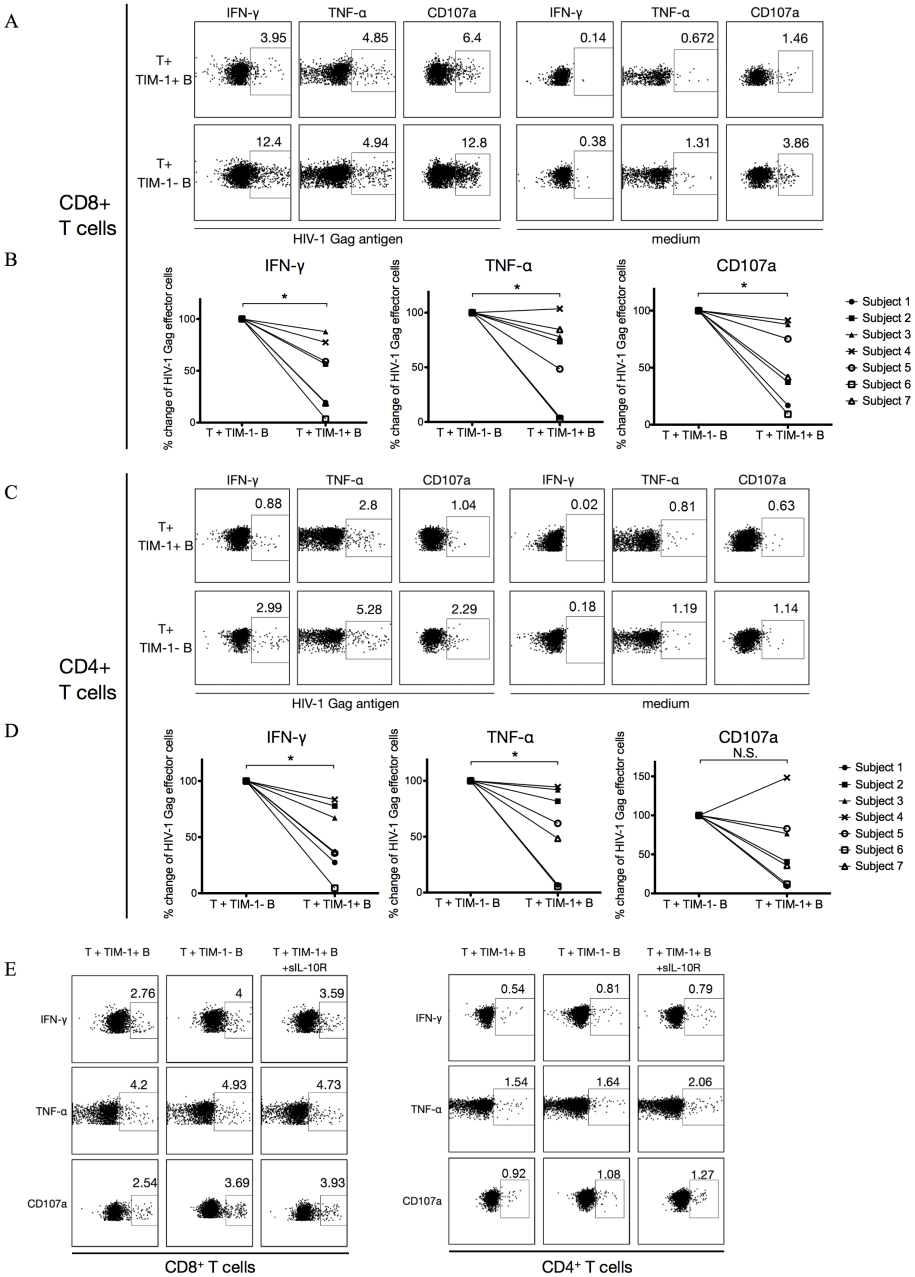
IL-10-producing B cells can suppress HIV-1-specific T cell responses. Purified T cells from seven HIV-1 EI individuals were cocultured at 1∶1 ratio with autologous purified TIM-1^+^ B cells or TIM-1^−^ B cells for 3 d and stimulated with HIV-1 Gag antigen (HIV-1 Gag Antigen) or without (Medium) during the last 6 h in the presence of GolgiStop and GolgiPlug. HIV-1 Gag-specific T cell cytokine production/degranulation was calculated as percentage of cytokine/degranulation positive T cells in T cells stimulated with HIV-1 Gag subtracting background from T cells cultured in medium. % change of HIV-1 Gag-specific T effector cells was calculated as 100% × (percentage of HIV-1 Gag-specific T effector cells from T cells co-cultured with TIM-1^+^ B cells ÷ percentage of HIV-1 Gag-specific T effector cells from T cells co-cultured with TIM-1^−^ B cells) (A) Flow cytometry dot plots of CD8^+^ T cell Gag responses from one representative HIV-1 EI individual. (B) Summary of CD8^+^ T cell responses from the seven HIV-1 EI subjects. (C) Flow cytometry dot plots of CD4^+^ T cell responses from one representative HIV-1 EI individual. (D) Summary of CD4^+^ T cell responses from the seven HIV-1 EI subjects. (E) IL-10-producing B cells suppressed HIV-1-specific T cell responses through IL-10. Purified T cells from HIV-1 EI individuals were cocultured with autologous purified TIM-1^+^ B cells with or without soluble IL-10 receptor (sIL-10R) or TIM-1^−^ B cells for 3 d. HIV-1 Gag antigen was added during the last 6 h in the presence of GolgiStop and GolgiPlug. Shown is a representative sample taken from three HIV-1 EI individuals. *: *P*<0.05. N.S.: non-significant (Wilcoxon matched-pairs test).

The direct correlation of IL-10-producing B cell frequency with viral load in EI suggests that IL-10-producing B cell may contribute to HIV-1 persistence in the early stages of infection, by suppressing immune responses as previously shown in other infections [37]. To determine if the IL-10-producing B cells in HIV-1 infected individuals exhibit regulatory function, we tested if these IL-10-producing B cells can suppress HIV-1-specific T cell responses. We isolated IL-10-producing B cells using TIM-1 as an IL-10-producing B cell marker. Purified T cells from untreated HIV-1 infected individuals were co-cultured with equal numbers of autologous purified TIM-1^+^ or TIM-1^−^ B cells for 72 h, and then stimulated with HIV-1 Gag antigen. Production of interferon-γ (IFN-γ) and tumor necrosis factor-α (TNF-α) and a degranulation marker, CD107a, of these T cells was then measured. As shown in Figure 5A and 5B, in all seven EI individuals tested, HIV-1-specific CD8^+^ T cell responses co-cultured with TIM-1^+^ B cells were suppressed compared with T cells co-cultured with TIM-1^−^ B cells (*P*<0.05). In order to determine the role of IL-10 in the IL-10-producing B cell-induced suppression, IL-10 blocking experiments using soluble IL-10 receptor (sIL-10R) were performed (Figure 5E). We showed reduction of suppression in three out of three HIV-1 EI individuals tested. Similarly, for HIV-1 Gag-specific CD4^+^ T cell responses, IFN-γ and TNF-α production were significantly suppressed by TIM-1^+^ B cells in all 7 EI individuals (*P*<0.05) while degranulation (CD107a) was suppressed by TIM-1^+^ B cells in 6 of the 7 subjects (Figure 5C and 5D). The suppression of HIV-1-specific CD4^+^ T cell IFN-γ and TNF-α responses could be rescued in three out of three HIV-1 viremic individuals by sIL-10R and degranulation (CD107a) in two out of three (Figure 5E). TIM-1^+^ B cells from CI individuals could also suppress autologous HIV-1-specific T cell responses (Figure S2). Taken together, these results indicated that IL-10-producing B cells detected in HIV-1 infected individuals can suppress HIV-1-specific T cell responses and this suppression is partially mediated by IL-10.

### Ex vivo IL-10-producing B Cell Frequencies Inversely Correlate with ex vivo HIV-1-specific T Cell Proliferative Responses

**Figure 6:**
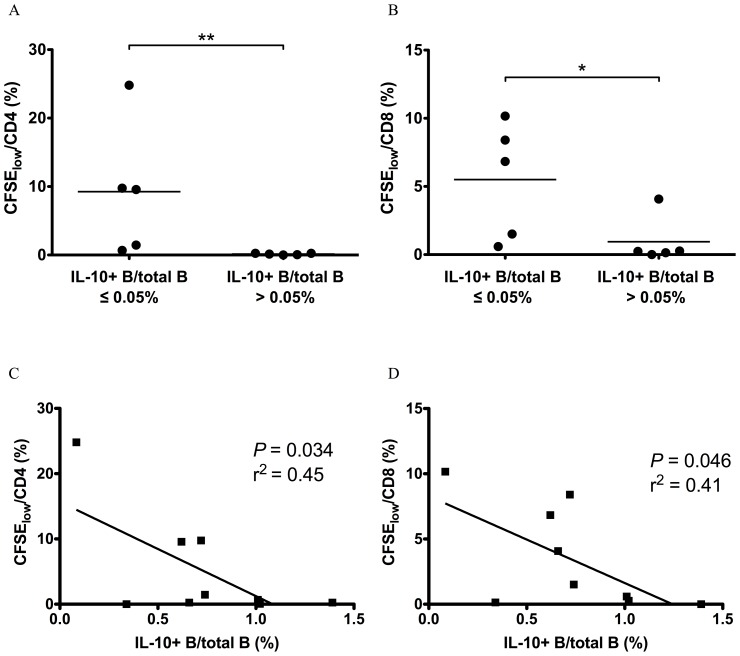
Correlation of IL-10-producing B cell frequency with HIV-1-specific T cell proliferation in early HIV-1 infection. CFSE-labeled PBMCs from ten HIV-1 EI subjects were stimulated with HIV-1 Gag pool for 6 d and proliferation of CD4^+^ and CD8^+^ T cells were analyzed with flow cytometry. These data were then correlated with contemporaneous IL-10-producing B cell frequencies in *ex vivo* unstimulated B cells or PMA/ionomycin stimulated B cells. (A) and (B) show proliferation of CD4^+^ and CD8^+^ T cells for the HIV-1 EI subjects grouped according to their IL-10-producing B cell frequency in unstimulated PBMC (IL-10-producing B cell frequency ≥0.05% or <0.05%). **: *P*<0.01. (Wilcoxon rank-sum test). (C) and (D) show correlation of frequency of IL-10-producing B cell in PBMC stimulated with PMA/ionomycin with proliferation of CD4^+^ and CD8^+^ T cells. Pearson’s correlation was used for correlation analysis.

HIV-1-specific T cell proliferation has been demonstrated to associate with and predict HIV-1 control [19], [47], suggesting that proliferation is important in suppressing HIV-1 replication. We therefore measured *ex vivo* HIV-1-specific T cell proliferation from EI individuals and compared them to contemporaneous frequencies of IL-10-producing B cells from the same blood sample. In EI, when *ex vivo* IL-10-producing B cell frequency was lower than 0.05% of total B cells, T cell proliferation in response to HIV-1 Gag antigen was easily detectable, but was undetectable when IL-10-producing B cell frequency was greater than 0.05% (Figure 6A and 6B). In addition, the HIV-1 Gag-specific proliferation of both CD4^+^ and CD8^+^ T cells inversely correlated with the frequency of IL-10-producing B cells detected after PMA/ionomycin stimulation (Figure 6C and 6D). Taken together, these results show that the frequency of peripheral blood IL-10-producing B cells is negatively associated with HIV-1-specific T cell proliferation.

### TLR Agonists can Promote Human IL-10-producing B Cell Differentiation

**Figure 7:**
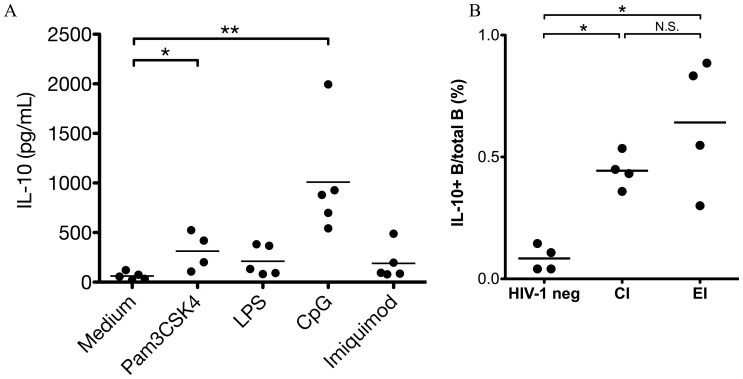
IL-10-producing B cell frequency is elevated by increased microbial translocation. (A) B cells were purified from PBMC of healthy subjects and then cultured in medium and treated with agonists for TLR-2 (Pam3CSK4), TLR-4 (LPS), TLR-7 (Imiquimod) and TLR-9 (CpG) for 12 h. IL-10 in the supernatant was measured. *: *P*<0.05. **: *P*<0.01 (Wilcoxon rank-sum test). (B) Single cells were prepared from sigmoid colons of healthy subjects, HIV-1 CI and EI individuals (four subjects for each group were tested) as described in Materials and Methods and cultured in medium in the presence of GolgiStop and GolgiPlug for 6 h. Cells were then stained with IL-10, CD3, and CD19 mAbs and analyzed with flow-cytometry. *: *P*<0.05. N.S.: non-significant (Kruskal-Wallis one-way ANOVA and Dunn’s test).

Since TLR agonists in blood and tissues are elevated in untreated HIV-1/SIV infection due to microbial translocation [48], we hypothesized that the increased level of IL-10-producing B cells in untreated HIV-1 infection could be induced by translocated TLR agonists. To test this, purified B cells from healthy donors were treated with agonists of TLR-2 (Pam3Cys-Ser-Lys4, Pam3CSK4), TLR-4 (lipopolysaccharide, LPS), TLR-7 (imiquimod) and TLR-9 (CpG), and IL-10 concentration in the supernatant was quantified. We found Pam3CSK4 and CpG alone could induce IL-10 production by purified B cells from healthy donors (Figure 7A). LPS and imiquimod tended to induce IL-10 production from purified B cells, but this was not statistically significant. Thus, some translocated microbial products can directly induce B cells to differentiate into IL-10-producing B cells in untreated HIV-1 infection via TLR-2 and/or TLR-9 signaling pathway.

### IL-10-producing B Cell Frequencies are Elevated in the Sigmoid Colon of Untreated HIV-1 Infected Individuals

The gut tissue is a major entry and replication site of HIV-1 [49]. Translocation of microbial product across the damaged gut mucosal barrier can occur early (14–28 days post infection) in the simian model of HIV-1 infection, which subsequently leads to elevated TLR agonist concentration in circulation [50]. To test if IL-10-producing B cells were also elevated in gut in HIV-1 infection, we detected IL-10-producing B cells in biopsies of sigmoid colon of untreated HIV-1 infected individuals and found that the frequencies of IL-10-producing B cell were also elevated in sigmoid colon of untreated HIV-1 infected individuals at both early and chronic stages, compared to healthy donors (Figure 7B).

## Discussion

In this study, we found IL-10-producing B cell frequencies elevated in peripheral blood and sigmoid colon of untreated HIV-1 infection. Moreover, we found IL-10-producing B cells from untreated individuals could suppress HIV-1-specific T cell responses in an IL-10-dependent manner. The frequency of IL-10-producing B cells directly correlated with viral load and inversely correlated with HIV-1-specific T cell proliferation in early infection. Together, these results suggest that IL-10-producing B cells in HIV-1 infection function as Bregs, similar to that previously described by others, in autoimmunity and other infections [2]–[5], [8], [11], [14], [51]. It is well known that HIV-1 can use multiple strategies to evade immune responses [25], [26], [44], [52]. Our findings here suggest that induction of IL-10-producing B cells can act as a potentially new viral evasion strategy of HIV-1. Elevated IL-10-producing B cell frequency is not a feature of all chronic virus infections, as we did not observe this in chronic untreated HCV mono-infection. During the preparation of this manuscript, Siewe et al. reported their findings on Bregs in HIV-1 infection [53]. Using CD19^+^CD24^hi^CD38^hi^ as a surrogate marker for Bregs, they found the frequency of CD19^+^CD24^hi^CD38^hi^ B cells in peripheral blood was actually decreased in HIV-1 viremic individuals compared with healthy donors, but the percentage of IL-10-producing B cells in the CD19^+^CD24^hi^CD38^hi^ B cell population after *ex vivo* stimulation was increased. In our study, we directly measured IL-10 production from B cells regardless of surface phenotype as a measure of IL-10-producing B cells, as previously shown by Das et al. [14]. Siewe also demonstrated that CD19^+^CD24^hi^CD38^hi^ cells from HIV-1 viremic individuals could suppress HIV-1-specific CD8^+^ T cell responses *in vitro* in an IL-10 dependent way and the frequency of these cells in peripheral blood correlated with higher viral load, immune activation, and PD-1 expression on CD8^+^ T cells. Together, their findings and ours support a role of IL-10-producing B cells in the dysfunction of HIV-1-specific CD8^+^ T cells. However, our findings are different from theirs in some aspects: we used IL-10 production to identify IL-10-producing B cells while they used CD24^hi^CD38^hi^, thus different populations may have been studied. As shown by previous reports, no surface markers have been found to be exclusively IL-10-producing Breg specific yet [37]. Thus, IL-10 production, together with demonstrating suppressive function, remains the most reliable marker to identify IL-10-producing Bregs [37]. We found IL-10-producing B cells from HIV-1 infected individuals detected directly *ex vivo* did not enrich in CD24^hi^CD38^hi^ B cells (Figure 3A). Consequently, we found the frequency of IL-10-producing B cells was increased in peripheral blood of HIV-1 viremic individuals while Siewe et al. reported the frequency of CD19^+^CD24^hi^CD38^hi^ Bregs decreased in peripheral blood of HIV-1 viremic individuals [53].

We investigated IL-10-producing B cells from HIV-1 infected individuals comprising different clinical stages. IL-10-producing B cell frequencies were elevated in EI and CI but not in LTNP. More importantly, IL-10-producing B cell frequency correlated best with viral load in EI but not in CI. Previous studies have shown HIV-1-specific T cell responses are important in controlling HIV-1 replication [19], [54]. IL-10-producing B cells could not only inhibit HIV-1-specific T cell responses *in vitro* but *in vivo* were associated with lower frequencies of HIV-1-specific T cell proliferative responses. Thus our findings indicate that IL-10-producing B cells are induced very early in infection and may have a role in T cell control of HIV-1 during this phase of the infection, possibly contributing to viral set-point establishment in chronic infection and resulting disease progression. Prospective studies in this regard are in progress to examine this possibility. Whether IL-10-producing B cell induction in untreated HIV-1 infection is also involved in suppression of HIV-1-specific antibody responses requires further study, but our findings here suggest that IL-10-producing B cells can suppress HIV-1-specific CD4^+^ T cell responses (Figure 5C and 5D), which are needed for providing B cell help.

The elevated IL-10-producing B cells in gut tissue from HIV-1 infected individuals reported here is interesting since gut is one of the main sites of HIV-1 replication and immune destruction early in the infection [49]. It was shown that microbes and/or microbial products can translocate from gut lumen to blood in HIV-1 infection and this translocation causes systemic immune activation, a hallmark of HIV-1 infection [48]. Many of these translocated microbes and/or microbial products are TLR agonists. TLR agonists were important signals promoting IL-10-producing Breg differentiation [37]. We also found that TLR agonists can directly promote IL-10 production of purified B cells from healthy donors. Thus, our findings suggest the elevation of IL-10-producing B cells in untreated HIV-1 infection may be induced, at least in part, by microbial translocation in the gut. Of note, HIV-1 replication is high in the gut and a potent mucosal T cell response is required for control. Further studies will be needed to determine whether gut IL-10-producing B cell negatively impact local gut viral control.

We found that IL-10-producing B cells from HIV-1 infected individuals were enriched in TIM-1^+^ B cells, similar to what was shown in mouse IL-10-producing Bregs in autoimmune diseases [41]. TIM-1 is a member of the T cell immunoglobulin domain and mucin domain (TIM) family and a costimulatory molecule for T cells and a cellular receptor for human hepatitis A virus [55]. Besides, TIM-1 was expressed on mouse germinal center B cells and was induced on mouse splenic B cells after BCR crosslinking; however, TIM-1 knockout mice had normal germinal centers and antibody response [56]. Ding et al. found more than 70% of mouse IL-10-producing Bregs were TIM-1^+^. Treatment of mouse with low affinity TIM-1 mAb RMT-10 could induce TIM-1^+^ IL-10-producing Bregs that were responsible for RMT-10 mediated allograft survival, suggesting that TIM-1 ligation can directly induce IL-10-producing Bregs [41]. Recently, Xiao et al. generated a TIM-1^Δmucin^ mouse line, which expressed TIM-1 at normal levels but without the extracellular mucin domain [42]. They found TIM-1^Δmucin^ mice had less IL-10-producing Bregs compared with WT mouse, especially in aging mice. Correspondingly, TIM-1^Δmucin^ old mice had more hyperactivated T cells and TIM-1^Δmucin^lpr mice developed more severe systemic autoimmunity than WT mice [42]. Therefore, TIM-1 engagement is vital for IL-10-producing Breg induction and maintenance in mice. Whether TIM-1 engagement is also important for IL-10-producing B cell differentiation in humans under physiological and pathological conditions, including HIV-1 infection, needs further study.

Surprisingly, IL-10-producing B cells were not enriched in the two B cell populations expanded in viremic HIV-1, i.e., the CD10 expressing transitional B cell population and the CD27^−^ exhausted B cells. IL-10-producing B cells tended to express CD27, consistent with either resting or activated memory phenotype.

IL-10-producing B cells in HIV-1 infection are enriched for B cells that are specific for HIV-1 Env, and gp140-specific B cells were enriched in IL-10-producing B cells. Thus these cells are likely induced in the vicinity of virus replication, possibly in the gut lymphoid tissue, which is a potent source of TLR ligands during HIV-1 infection. Since other signals, including BCR activation, have also shown to promote IL-10-producing Breg differentiation [8], [37], HIV-1 Env itself could potentially induce IL-10-producing B cells directly through BCR stimulation. The recent work by Moir et al. demonstrated that exhausted B cells are enriched for HIV-1 specificity [44]. Given, that the IL-10-producing B cell population represents a minor B cell population, our data are still consistent with a major percentage of HIV-1-specific cells to be found within the exhausted B cell population.

Our data showed that the suppression of HIV-1-specific responses mediated by IL-10-producing B cells was dependent on IL-10. However, other mechanisms such as cell-cell contact cannot be excluded. Blair et al. found that the suppression of IFN-γ production of CD4^+^ T cells by human IL-10-producing Bregs was completely rescued when they combined antibodies blocking IL-10, CD80, and CD86, suggesting that the CD80/CD86 costimulation signaling pathway participates in the IL-10-producing Breg-mediated suppression of T cell responses [1]. Further studies are needed to determine whether CD80/CD86 and other costimulation molecules expressed by IL-10-producing B cells are involved in suppressing of HIV-1-specific T cell responses.

Based on the above findings it is compelling to suggest that HIV-1 can directly induce IL-10-producing B cells early in infection to subvert effective T and B cell immune responses. It is unclear whether such an effect is direct, or secondary to microbial translocation due to gut CD4^+^ T cell depletion seen early in infection. However, it is also possible that IL-10-producing B cells are induced as a reaction to extensive immune activation, so characteristic of HIV-1 infection. Since early HIV-1 infection is associated with dramatic CD8^+^ T cell activation and expansion, it is possible that IL-10-producing B cells play a physiologic role in preventing CTLs from killing B cells that are maturing to mount neutralizing antibody responses. Future work correlating IL-10-producing B cells with development of neutralizing antibody responses is required to address this. Also, IL-10-producing B cells could play a role in downregulating overall immune activation that could fuel further HIV-1 replication. Since we did not observe positive correlation between IL-10-producing B cell frequency and viral load in chronic established untreated infection, these opposing roles of IL-10-producing B cell function (preventing immune control versus dampening immune activation) could be at play.

## Supporting Information

Figure S1 **Correlation of IL-10-producing B cell frequency with HIV-1 plasma viral load and CD4 T^+^ cell count in untreated chronic HIV-1 infection.** Correlation of IL-10-producing B cell frequency in (A) *ex vivo* unstimulated PBMC and (B) PBMC stimulated with PMA/ionomycin for 5 h with viral load. Correlation of IL-10-producing B cell frequency in (C) unstimulated PBMC and (D) PBMC stimulated with PMA/ionomycin for 5 h with CD4^+^ T cell count. Ten HIV-1 CI subjects were tested. Pearson’s correlation was used for correlation analysis.
(TIFF)

Figure S2 **IL-10-producing B cells can suppress HIV-1-specific T cell response in chronic infection.** Flow cytometry dot plots of T cell responses from one representative chronic HIV-1 individual. Purified T cells from chronic viremic HIV-1 infected individuals were cocultured with autologous purified TIM-1^+^ B cells or TIM-1^−^ B cells for 3 d and stimulated with HIV-1 Gag antigen during the last 6 hours in the presence of GolgiStop and GolgiPlug.
(TIFF)
