# Supplementary figures and images for: IL-10-Producing B Cells Are Induced Early in HIV-1 Infection and Suppress HIV-1-Specific T Cell Responses
Source: PLoS One. 2014 Feb 21;9(2):e89236. doi: 10.1371/journal.pone.0089236 (PMC3931714; doi:10.1371/journal.pone.0089236)

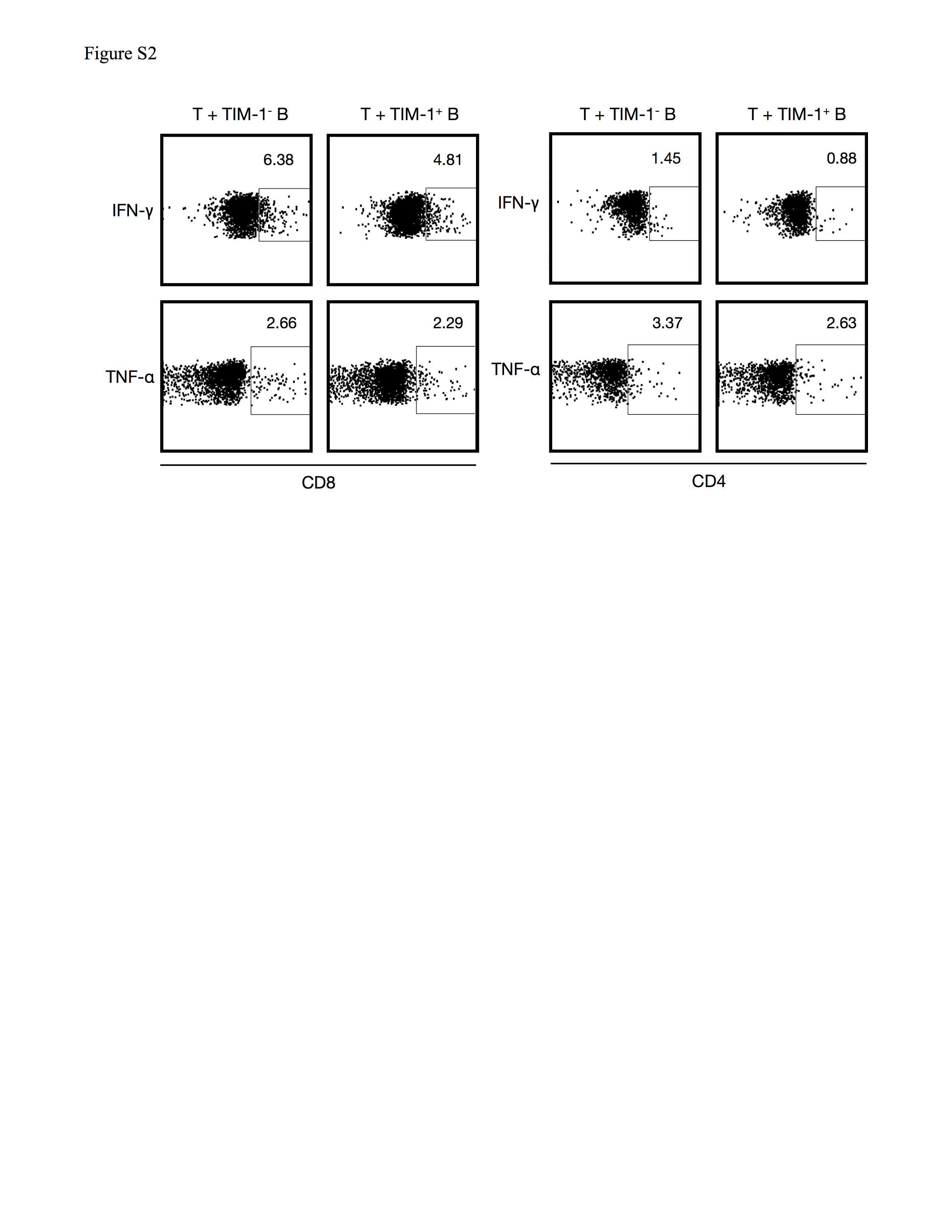

Supplement: Figure S2 — IL-10-producing B cells can suppress HIV-1-specific T cell response in chronic infection. Flow cytometry dot plots of T cell responses from one representative chronic HIV-1 individual. Purified T cells from chronic viremic HIV-1 infected individuals were cocultured with autologous purified TIM-1+ B cells or TIM-1− B cells for 3 d and stimulated with HIV-1 Gag antigen during the last 6 hours in the presence of GolgiStop and GolgiPlug. (TIFF) [file pone.0089236.s002.tiff]
